# Identification of circular RNA hsa-PHACTR4_0009 as a new class of biomarker for transposition of the great arteries^☆^

**DOI:** 10.1016/j.jmccpl.2025.100830

**Published:** 2025-11-23

**Authors:** Neha Rawal, Anmol Gurgela, Snigdha Kumari, Manoj Kumar Rohit, Ajay Bahl, Anupam Mittal

**Affiliations:** a Department of Translational and Regenerative Medicine, PGIMER, Chandigarh, India; b Department of Obstetrics & Gynaecology, PGIMER, Chandigarh, India; c Department of Cardiology, PGIMER, Chandigarh, India

**Keywords:** Transposition of great arteries, Circular RNA, Whole transcriptomics, Biomarker

## Abstract

Dextro-Transposition of great arteries (d-TGA) is a cardiac birth anomaly with reversed anatomic position of the aorta and pulmonary artery, with a poor prognosis. The molecular aetiology of d-TGA remains elusive, possibly due to polygenic contributions and gene-environment interactions, suggesting the role of epigenetic components in d-TGA. Various non-coding RNAs, like circular RNA (circRNA), epigenetically regulate gene expression and may also serve as putative biomarkers because of their exceptionally high stability in plasma. In this study, we performed whole transcriptome analysis in the plasma of diseased and healthy neonates to identify circular RNA specifically dysregulated in the d-TGA condition. Our data suggested that a circular RNA, i.e., hsa-PHACTR4_0009, is significantly increased in the plasma of d-TGA neonates and has 85 % sensitivity and 80 % specificity with an AUC value of 0.900 with foetal echocardiography as the gold standard. Interestingly, d-TGA cases had a proportional increase of hsa-PHACTR4_0009 with the severity of the disease, as evident by comparably high levels in patients with intact interventricular and interatrial septa. Transcriptomics analysis further indicated that there is a dysregulation of the focal adhesion assembly genes like vimentin, profilin, gamma-actin and emerin, corroborating with hsa-PHACTR4_0009 expression. Notably, overexpression of hsa-PHACTR4_0009 led to increased cell migration in H9C2 cells, suggesting that defects in cell migration are involved in the development of d-TGA. Collectively, our study suggests hsa-PHACTR4_0009 as a potential biomarker of d-TGA and provides a highly valuable insight into the molecular pathogenesis of d-TGA from an unexplored perspective, enabling a better prognosis for these patients.

## Graphical abstract

**Figure:**
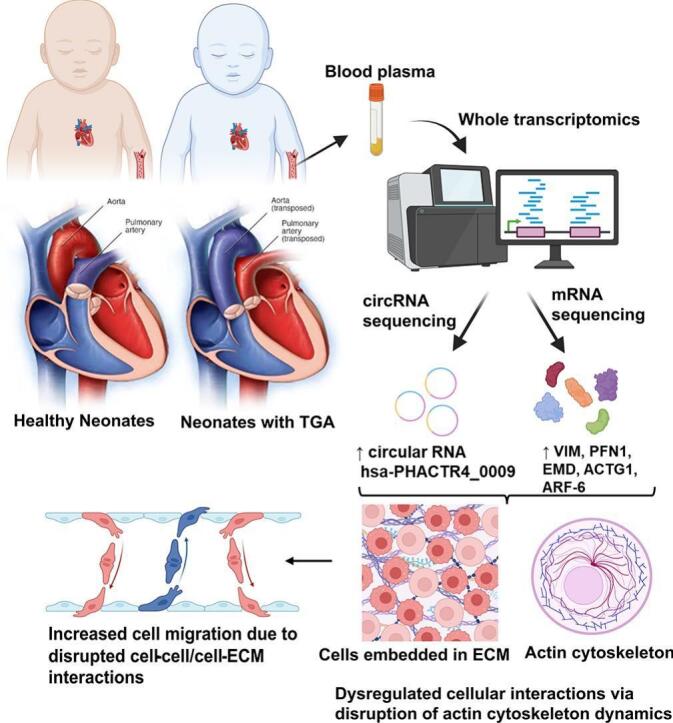

## 1 Introduction

d-TGA is a complex congenital cardiac defect, constituting 3 % of all congenital heart diseases (CHDs) and 20 % of all neonatal cyanotic cardiac abnormalities [1]. The incidence of d-TGA is 4.7 per 10,000 live births, with prenatal detection rates below 50 % [2]. d-TGA is described in a variety of different ways, but the most precise representation of d-TGA is a congenital disability where the pulmonary artery emerges from the morphological left ventricle (mLV) while the aorta emerges from the morphological right ventricle (mRV), called dextro-TGA (d-TGA). This arrangement causes the incoming oxygen-poor blood through the vena cavae to recirculate through the body via the aorta, while the oxygenated blood coming from the lungs shunts back to it via the pulmonary artery and leads to severe deficiency of oxygen, resulting in cyanosis in the body [3]. In this condition, the prognosis of the paediatric patient is bad, and survival is minimal. A comprehensive analysis of the disease reveals that it frequently occurs with the associated lesions like ventricular septal defect (VSD) and atrial septal defect (ASD) [4]. Until a final surgical correction is made, these concurrent abnormalities serve as vital compensatory mechanisms that allow the mixing of oxygenated and deoxygenated blood to maintain tissue perfusion and systemic oxygenation [5]. Researchers have suggested a complex polygenic aetiology and the role of epigenetic factors in the development of abnormalities in the great arteries. However, identifying the main issue in congenital heart abnormalities can be difficult due to the complexities within the developing heart. Researchers have explored various non-coding RNAs (ncRNAs) like miRNAs and lncRNAs, which may epigenetically control the developmental defects in the heart [6,7] but no study has explored the role of circular RNA in TGA. Circular RNA (circRNA), a type of non-coding RNA, has higher stability in blood than other ncRNAs due to covalently linked 5′ and 3′ ends of circular RNA, which renders them resistant to exonuclease action [8]. These circRNAs may serve as potential biomarkers of various diseases by virtue of this property. Many circRNAs have been investigated as biomarkers of various cardiovascular diseases, but none have been reported to be associated with TGA [[9], [10], [11]].

This study focuses on identifying circular RNA specifically dysregulated in the TGA neonates. Our study elucidates a dysregulated circRNA, hsa-PHACTR4_0009, which might play an essential role in epithelial-to-mesenchymal transition (EMT) as it positively upregulates cell migration. These findings may improve the postnatal management strategies for TGA neonates. Besides, hsa-PHACTR4_0009 might play a crucial role in diagnosing d-TGA as a putative biomarker in the prenatal stages.

## 2 Materials & methods

### 2.1 Study design and participants

**Fig. 1:**
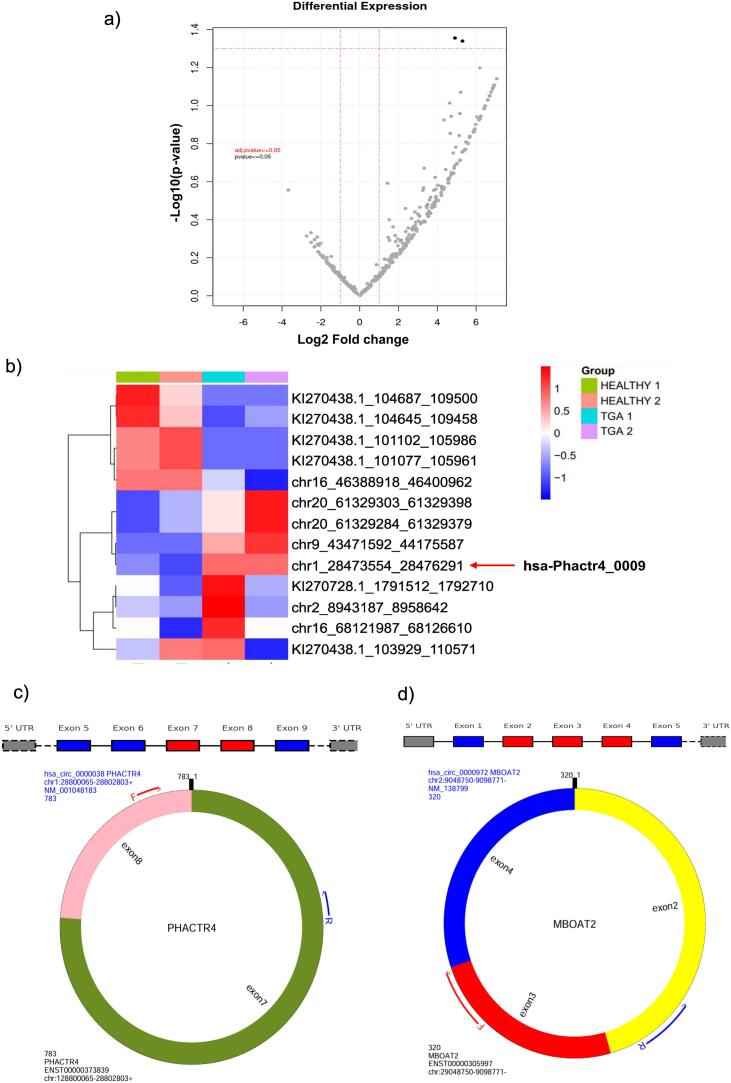
Differentially expressed circRNA in TGA patients (a) A volcano plot representing dysregulated circRNA in neonates with TGA compared to healthy neonates (*n* = 2) (b) Heat map representing distinct expression profiles of circular RNA in healthy vs diseased subjects. (c) Biogenesis of hsa-PHACTR4_0009 made up of 783 bp from exons 7 & 8 of the parent gene. (d) Secondary structure of hsa-PHACTR4_0009 as derived from circAtlas v 3.0.

This study used paired cases and controls of TGA patients to identify dysregulated genes that can be later used to predict their role in diagnosing these congenital disabilities (Fig. 1). Twenty-two paediatric patient samples of the d-TGA cohort were collected from the Advanced Paediatric Centre, PGIMER, Chandigarh. Neonates with other congenital disabilities or genetic syndromes were excluded from the discovery phase of the study. Twenty-two healthy neonates were recruited as a control group and were matched with cases based on age and gender to reduce the heterogeneity due to these factors. Out of these, two samples from each group were used for transcriptomic profiling and for the validation phase, we recruited 20 paediatric patients with d-TGA and 20 healthy neonates to confirm the expression profiles of dysregulated circular RNA through qRT-PCR. Control group samples were recruited during routine biochemistry and haematology testing, provided the patient had a mild illness, such as fever, viral infection, etc.

Written informed consent was taken for all the samples, and sampling was done with the approval of the institutional ethics committee.

### 2.2 RNA extraction from blood plasma

1 ml of blood from each healthy and diseased neonate was collected in an EDTA-anticoagulant vial and centrifuged at 2000 rpm for 10 min. The upper layer of plasma was isolated in RNase & DNase-free cryovials and stored at −80 °C for later use. According to the user manual, forty-four samples were processed for total RNA isolation from the blood plasma using the MagMAX™ RNA isolation kit (cat no #AM1939, Applied Biosystems), and quantity was measured using Qubit® 4.0 fluorometer.

### 2.3 Preparation of the library

The RNA-seq library for paired-end sequencing was manufactured using the NEBNext® Ultra™ RNA Library Prep Kit for Illumina (NEB #E7770) with an input of 5–10 ng RNA as per the manufacturer's instructions. A human-specific ribosomal RNA-depletion kit was used along with RNaseH and DNaseI treatment to remove ribosomal RNA. Ribosomal-depleted RNA was fragmented, underwent first & second-strand cDNA synthesis, end-repair, 3′ adenylation, adapter ligation, and selective enrichment of adapter-ligated DNA fragments through PCR amplification, followed by validation of the prepared Library on an Agilent 4150 tape station. The prepared library was pooled with other samples, denatured & loaded onto the flow cell. Cluster generation & sequencing were conducted using the Illumina Novaseq 6000 platform to generate 2 × 150 bp paired-end (PE) reads to generate ∼12GB of data/Sample. The amplified libraries were analysed on TapeStation 4150 (Agilent Technologies) using RNA ScreenTape® as per the manufacturer's instructions.

### 2.4 Bioinformatics Analysis

Raw data generated by Illumina Novaseq was processed for adapter and low-quality data removal using Trimmomatic software version 0.39. The complete process was divided into three stages:

a.) The Alignment of reads to the reference genome.
  A star mapper aligned all sample reads with the reference genome. The reference genome of humans and its GTF file were downloaded from UCSC (University of California, Santa Cruz).
b.) Parsing for analysis
  This step parses fusion junction information from the results of the STAR aligner to prepare the necessary files for the subsequent analysis.
c.) Circular RNA identification using DCC.
  The differential expression of circular RNA was predicted using the DCC. This tool uses the STAR read mapper output to detect the back-splice junctions. The first step is to align reads using the STAR aligner against the hg38 reference genome from GENCODE, along with the reference GTF. This step generated a “chimeric out junction,” containing chimerically aligned reads, including circular RNA and spanning reads.

### 2.5 Differential expression of circular RNAs

The biological replicates were grouped as Control and Treated for differential expression analysis. For differential expression analysis, the DESeq2 v1.34.0 package was used. Transcripts with fewer than five reads in patient samples were excluded from further analysis. The differential expression analysis was subsequently conducted on the variance-stabilised normalised counts. The identified circular RNA expressions were characterised using the DESeq2 R package. The circular RNA expression with a *p*-value ≤0.05 is considered significant. The logFC value >1 is regarded as upregulation, and <−1 is considered downregulation. The significantly expressed genes are further annotated using CircFunBase [12].

### 2.6 Gene ontology (GO) analysis

The Gene Ontology project offers controlled vocabularies of defined terms indicating gene product properties. GO was assigned to significantly differentially expressed transcripts using OmicsBox.

### 2.7 Pathway analysis

Ortholog assignment and mapping of differentially expressed isoforms of CircRNAs to the biological pathways were performed using the KEGG automatic annotation server (KAAS). Isoforms associated with all 3 DGE combinations were searched individually against the KEGG database using BLASTX with a threshold bit-score value of 60 (default). In addition, 4099 unique isoforms were also compared using the same parameters.

### 2.8 circRNA expression by qRT-PCR

For the replication phase, the expression of identified circRNA was analysed in a larger cohort of neonates with TGA at the early stages of pregnancy. cDNA synthesised using QuantiTect Reverse Transcription Kit (Cat. No. / ID: 205313, Qiagen) per the manufacturer's protocol. qRT-PCR was performed using circular RNA-specific divergent primers designed using circInteractome [13] and circPrimer2.0 [14]. The data were normalised to GAPDH RNA as an internal control.

**Table:** 

Name	Forward Primer	Reverse Primer
hsa-circPHACTR4	CACAGTGTCTACGGGAGGAA	GGGTGGGGACGGTTTTGATA
hsa-circMBOAT4	TCATAGGAGTGGAGAACATGCA	AGTGCAAGATAAAGGCCCAA
hsa-GAPDH	GAGTCCACTGGCGTCTTCA	GGTCATGAGTCCTTCCACGA

### 2.9 Principal component analysis and data visualisation

Data was visualised using GraphPad Prism version 8. PCA, heatmaps, and volcano plots were generated in R. Correlations were tested using Pearson correlation coefficients.

### 2.10 Plasmid construction and transfection

As per the manufacturer's instructions, cDNA (Cat. No. 205313, Qiagen) was prepared from the RNA isolated from human PBMCs by the Trizol method. The circ-PHACTR4 over-expression vector was created by cloning PHACTR4 cDNA into the ATP1-ZKSCAN-spGFP-Blstn vector (sourced from Florian Karreth, Moffitt Cancer Centre, Florida 33612, USA) in between the *Sna*BI and *Pst*I restriction sites. Lipofectamine 3000 (Invitrogen, cat. 100022050) was used for transfection of the overexpression vector along with the vector control in H9C2 cells for 24 h, as per the manufacturer's protocol.

### 2.11 Cell culture and scratch assay

H9C2 cells were cultured in Dulbecco's Modified Eagle's Medium (DMEM, MP Biomedicals cat. 091233354) supplemented with 10 % heat-inactivated foetal bovine serum (FBS, Gibco, Cat.) at 37 °C with 5 % CO2 in an incubator. Cells from a 25 cm^2^ flask that had been grown to full confluency (∼2.8–3.0 × 10^6^) were reseeded into a six-well flat-bottom plate (NEST, Cat. No.703001) for the wound healing assay, at a seeding density of 2.0–3.0 × 10^5^ cells per well. After 24 h, uniform cross-shaped scratches were created using a sterile 200-μl pipette tip in each well. Long-term observation was done at 0 h, 6 h, 12 h, 24 h and 48 h at the microscope stage for live cell imaging. Wound Healing Analysis was done by Image J software.

### 2.12 Statistical analysis

Statistical data analysis was performed after data normalization, using a non-parametric paired Wilcoxon signed-rank test to identify circular RNAs differentially expressed between TGA neonates and healthy neonates. An unpaired *t*-test was used for overexpression studies. Wound healing assay data were analysed using two-way ANOVA with Sidak's post hoc correction for multiple comparisons between two groups at different time points.

## 3 Results

### 3.1 Baseline attributes of control and d-TGA patient plasma samples

Baseline characteristics of d-TGA patients, such as age and sex, are mentioned in Table 1. Consistent with the reported literature, our study population had a higher male-female ratio affected with TGA [15]. Moreover, TGA neonates showed significantly lower oxygen saturation (SpO2) and a drop in systolic and diastolic blood pressure. Interestingly, TGA patients had higher haemoglobin levels than the control group. In addition, most of the cases of TGA were found to be associated with other cardiac lesions like ASD (Atrial septal defect), VSD (Ventricular septal defect), and PDA (Patent ductus arteriosus). However, there was no significant effect on the birth weight of TGA and healthy neonates.

**Table 1:** Characteristics of recruited subjects (patients with TGA and controls). Median values were used for comparison. *Non-parametric unpaired Mann-Whitney *U* test. **Non-parametric paired Wilcoxon signed-rank test.

Variables	Neonates with TGA (*n* = 22)	Healthy Neonates (n = 22)	p-Value
1. Age (days)	12	3	
2. Sex (Male)	18/22	12/22	
3. Birth Weight (kg)	2.75 (2–3.5)	2.424 (1.9–3.2)	0.0529
4. SpO2 (mmHg)	**71** **%** (35–89 %)	98 % (95–99 %)	<0.0001^⁎⁎⁎^
5. Haemoglobin (gm/dL)	**13.85** (9.1–22)	11.5 (8.9–13.7)	0.0014^⁎⁎^
6. ASD	16/23	N/A	
7. VSD	13/23	N/A	
8. PDA	14/23	N/A	
9. Systolic BP (mmHg)	**79** (66–91)	120	<0.0001^⁎⁎⁎^
10. Diastolic BP (mmHg)	**44.5** (27–69)	80	<0.0001^⁎⁎⁎^

Bold here represents the significantly different characteristics between the two groups (TGA vs Control) that leads to important interpretations of the study as discussed in the 'Discussions' part.

### 3.2 Differentially regulated circular RNA in d-TGA neonates

Whole transcriptomics with circRNA enrichment analysis yielded dysregulated circRNAs and genes. Using CIRCexplorer2v2.3.8, alignment and annotation of circular RNA were done, and the workflow pipeline to predict circular RNA is represented in Fig. S1. PCA plot revealed 25.82 % of total variance in the first principal component (PC1) and 39.12 % of total variance in the second principal component (PC2) (Fig. S2). This indicates that control and d-TGA samples were clustered in PC1, showing similar impacts and indicating their intra-group resemblance. Moreover, PC2 revealed specific differences in the circular RNA expression profiles, indicating inter-group differences between the control and d-TGA samples.

Circular RNA-seq analysis identified 48,940 differentially expressed transcripts in d-TGA neonates compared to control neonates. The upregulated top-hits were selected based on log2 fold-change value ≥1, as represented in the volcano plot (Fig. 1a). The heat map of the top upregulated circular RNA in the blood of d-TGA neonates compared to the control group (Fig. 1b). We selected two significantly upregulated circRNAs, i.e., hsa-PHACTR4_0009 and hsa-MBOAT2_0001, based on *p*-value <0.05 for validation in a larger cohort. The details of these two circular RNAs, such as the fold change in the d-TGA group, their chromosomal location and the parent gene from which they are derived, are given in Table 2. hsa-PHACTR4_0009 is derived from back-splicing of the exon 7 and exon 8 of the PHACTR4 gene located on chromosome 1. It is made up of 783 bp (Fig. 1c), while hsa-MBOAT2_0001 is composed of 320 bp derived from circularisation of exons 2, 3 and 4 of the MBOAT2 gene located on chromosome 2 (Fig. 1d). Using the circAtlas v3.0 database [16] we retrieved other information about these circRNAs, such as their registered ID, sequences, type of circular RNA, multiple conservation scores, junction ratio, and expression in different tissues (Table 3).

**Table 2:** Details of the upregulated circular RNA as evident from RNA-seq results.

Sr. No.	Chromosomal Location	Genomic position	Log2 fold change	P-value	Host gene
1	Chromosome 1	28473554_28476291	5.29	0.045	ENSG00000204138.12
2	Chromosome 2	8943187_8958642	4.90	0.044	ENSG00000143797.11

**Table 3:** Characteristics of upregulated circular RNA as depicted by circAtlas v3.0.

Sr. No.	CircAtlas ID	Mature Length	Multiple Conservation Score	Strand	Type of circular RNA
1.	hsa-PHACTR4_0009	783	2.66667	+	Exonic
2.	hsa-MBOAT2_0001	322	8.81818	−	Intronic

### 3.3 hsa-PHACTR4_0009 expression was upregulated in d-TGA cases and is associated with disease severity

**Fig. 2:**
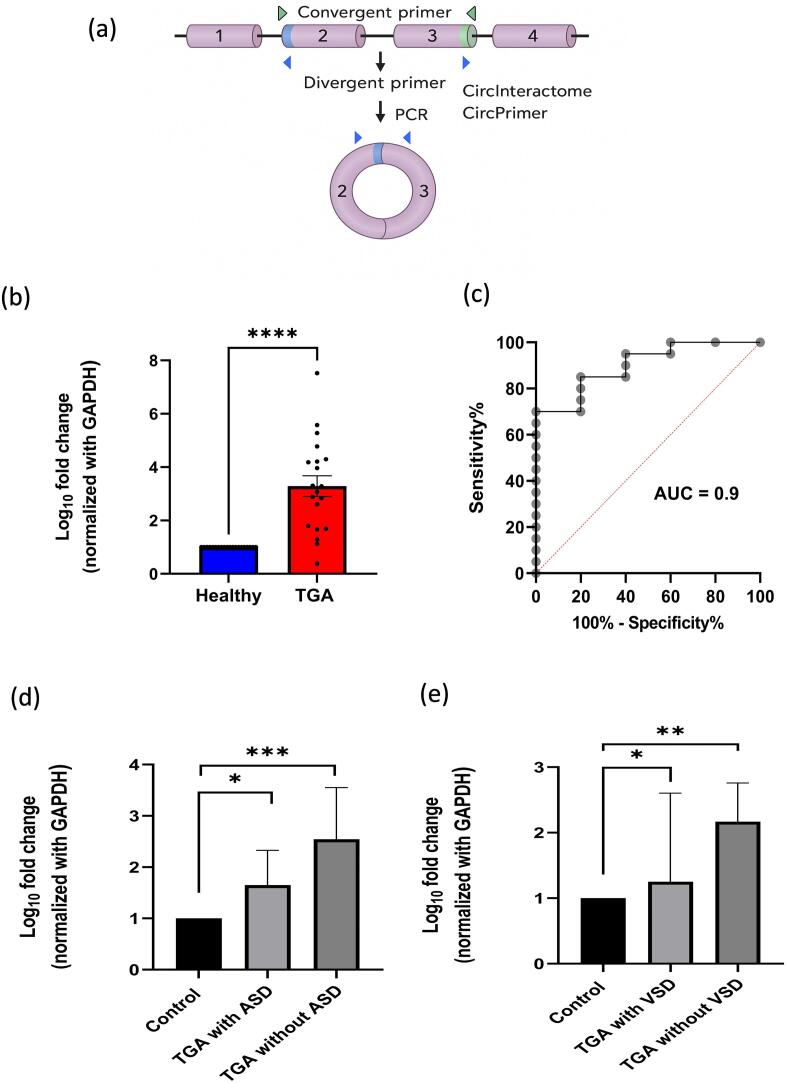
ROC curve and Expression profile of hsa-PHACTR4_0009 (a) Divergent primers were designed using circInteractome and circPrimer to detect the circular transcript of PHACTR4 specifically. (b) qRT-PCR results showing upregulated levels of hsa-PHACTR4_0009 in TGA cases compared to matched controls (*n* = 20, *p*-value =0.0003, non-parametric paired Wilcoxon signed-rank test). (c) ROC curve of hsa-PHACTR4_0009 (p-value = 0.0066) (d & e) Expression levels of hsa-PHACTR4_0009 in TGA cases associated with ASD and VSD, respectively. Data are represented as mean fold change (2^-ΔCt) of TGA compared to controls. GAPDH was used as an endogenous control for normalization. Statistical significance was defined as *P < 0.05; **P < 0.01; ***P < 0.001.

To validate the expression of the significantly upregulated circular RNA in a larger cohort of d-TGA paediatric patients (n = 20) compared to the control group of neonates (*n* = 20), we adopted qRT-PCR and designed divergent primers to detect the expression levels of circular RNA specifically, but not their linear counterparts. We used circInteractome and circPrimer v2.0 to precisely create the divergent primers against the junction region of circularRNA (Fig. 2a). qRT-PCR results revealed the significant upregulation of hsa-PHACTR4_0009, corroborating our circRNA sequencing data (Fig. 2b). However, due to poor qPCR amplification specificity of hsa-MBOAT2_0001 during standardisation, we omitted MBOAT2_0001 for further analysis. Additionally, the host gene of hsa-MBOAT2_0001 is a lower-priority candidate for validation because it does not exhibit strong associations with cardiac development. Therefore, we selected hsa-PHACTR4_0009, but not hsa-MBOAT2_0001, as a potential candidate to be established as a biomarker for d-TGA. To explore the potential source of hsa-PHACTR4_0009, we queried publicly available circRNA databases like circAtlas v3.0. Our in-silico analysis indicates that the expression of PHACTR4-derived circRNAs, including hsa-PHACTR4_0009, was highly detectable in the heart and blood with 0.10525 and 1.771 counts per million circular transcripts. This is supported by the data of junction ratio of hsa-PHACTR4_0009 in the heart tissue and blood, viz. 0.571 and 0.584, respectively. Protein Atlas revealed that the host gene of hsa-PHACTR4_0009 is expressed mainly in heart endothelial cells, and some expression is detected in cardiomyocytes as well. These findings indicate that hsa-PHACTR4_0009 has expression in cardiac tissue.

Next, we plotted the receiver operating characteristics (ROC) curve to check the diagnostic ability of hsa-PHACTR4_0009. At a threshold value of 0.01042, the hsa-PHACTR4_0009 has 85 % sensitivity and 80 % specificity, with a likelihood ratio of 4.250, with the area under the curve (AUC) value of 0.900 (Fig. 2c). Furthermore, we computed the association of the expression profile of hsa-PHACTR4_0009 with the disease severity, which heavily depends on the adequacy of atrial/ventricular level shunting via an ASD/VSD, respectively. The data demonstrated that the expression of hsa-PHACTR4_0009 was significantly increased with an intact atrial/ventricular septum, indicative of inadequate mixing between deoxygenated and oxygenated blood and the presence of severe cyanosis in d-TGA patients (Fig. 2d-e). These findings suggest that hsa-PHACTR4_0009 is increased in d-TGA congenital disease and is directly associated with the severity of the d-TGA condition.

### 3.4 Abnormalities in cell migration underlie d-TGA development and progression

**Fig. 3:**
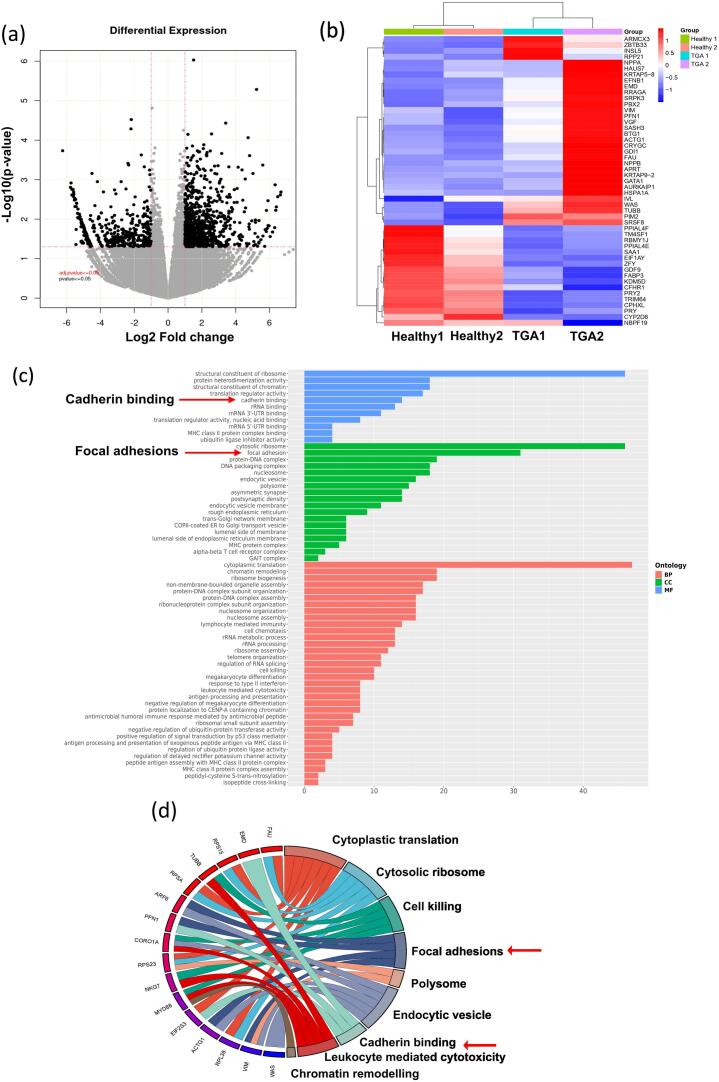
Transcriptomic changes associated with hsa-PHACTR4_0009 in d-TGA. (a) A volcano map illustrating the differentially expressed protein-coding genes in d-TGA patients than in healthy controls. (b) Heatmap depicting substantially dysregulated genes expressed across the two groups. (c) Gene Ontology (GO) enrichment analysis demonstrating altered biological pathways in d-TGA. (d) Chord diagram illustrating the connections between dysregulated genes and the corresponding GO terms.

To better comprehend the molecular mechanisms associated with developing d-TGA, we utilised RNA-seq data to visualise the differentially expressed genes in d-TGA cases compared to matched controls. A total of 1277 genes were identified as significantly dysregulated between d-TGA neonates and matched controls, out of which 862 were upregulated and 415 were downregulated, based upon a *p*-value <0.05 and the log2fold change >1 and < 1 in either direction (i.e., increased or decreased expression levels). Volcano plot of dysregulated genes and the representative heatmap of top 50 DEGs (Fig. 3a-b) shows a distinct expression profile of various protein-coding genes in healthy and TGA patients. Next, we conducted gene ontology enrichment analysis to identify the plausible role of DEGs in the pathogenesis of d-TGA. The results revealed that the predominantly associated terms in GO were linked to focal adhesions and cadherin binding in the cellular component and molecular functions categories, respectively (Fig. 3c). Our data showed increased Vimentin (VIM), Profilin (PFN1), and Gamma-actin (ACTG1), ADP ribosylation factor 6 (ARF-6) in TGA as compared to the control group; these are all mediators of focal adhesion and are reported to control cytoskeletal motility and migration (Fig. 3d). Similarly, the GO term ‘cadherin binding’ revealed the upregulation of Emerin (EMD) in d-TGA cases (Fig. 3d), a key protein located in the inner nuclear envelope and crucial for cell migration [17]. Collectively, these results signify that defects in the expression of cell adhesion molecules cause dysregulated cell migration, which underlies cardiac development, leading to the pathogenesis of d-TGA.

### 3.5 hsa-PHACTR4_0009 overexpression leads to an increase in cell migration

**Fig. 4:**
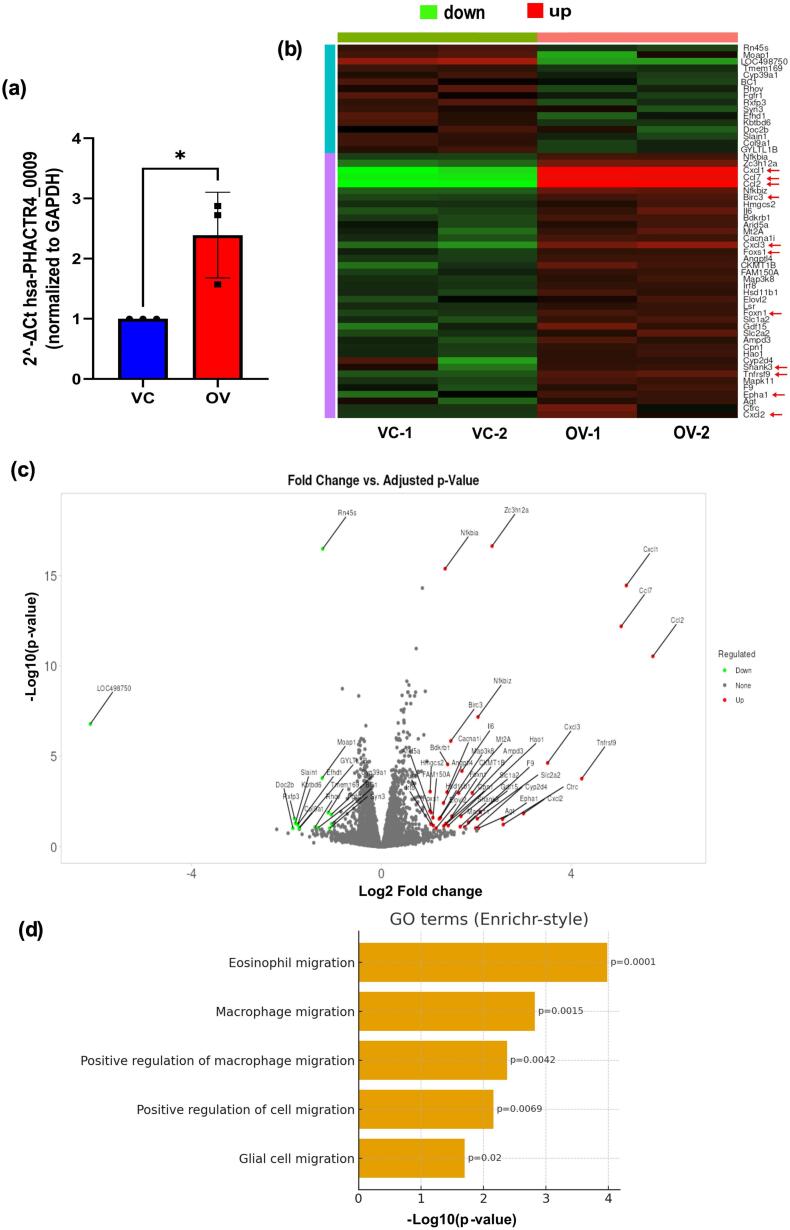
hsa-PHACTR4_0009 overexpression upregulates migration-associated gene programs. (a) Verification of increased expression of hsa-PHACTR4_0009 upon transfection with an overexpression vector (VC = Vector control, OV = hsa-PHACTR4_0009 overexpression**,***n* = 3, p-value 0.0277, unpaired *t*-test). (b-c) A heatmap and volcano plot depicting dysregulated genes in hsa-PHACTR4_0009 overexpression compared to the vector control. (d) Gene Ontology enrichment analysis of the RNA-seq data depicting significant upregulation of migration-related processes upon hsa-PHACTR4_0009 overexpression.

In order to investigate the functional role of hsa-PHACTR4_0009 in cardiomyocytes, we cloned the PHACTR4 gene into an expression vector to promote hsa-PHACTR4_0009 overexpression. At the RNA level, successful overexpression was verified in the H9C2 rat cardiomyocyte cell line (Fig. 4a). The global transcriptional alterations linked to hsa-PHACTR4_0009 upregulation were then evaluated by RNA sequencing and heatmaps and volcano plots of all the DEGs were plotted (Fig. 4b, c). When circ-PHACTR4–overexpressing cells were compared to vector controls, gene ontology analysis of the differentially expressed genes revealed a significant upregulation of cell migration-related processes (Fig. 4d).

**Fig. 5:**
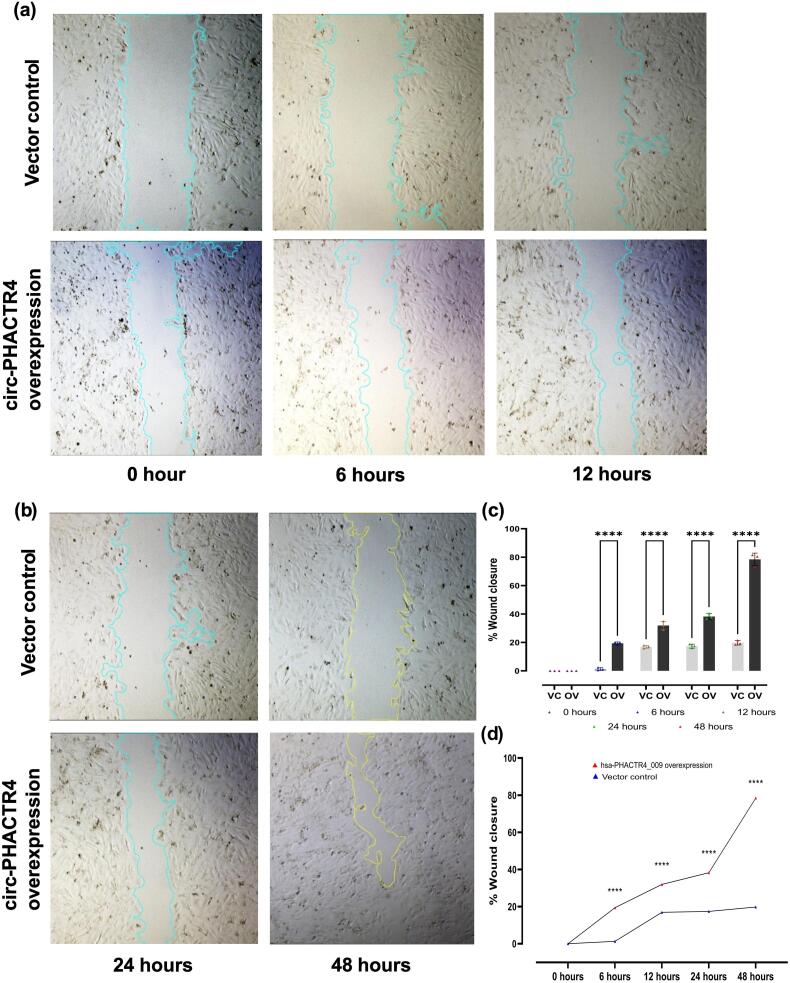
Functional characterisation of hsa-PHACTR4_0009 in H9C2 cardiomyocytes. (a-b) Representative images from wound healing tests showing that hsa-PHACTR4_0009 overexpressing cardiomyocytes exhibit accelerated scratch closure than controls at the indicated time points. (c-d) Quantitative evaluation of wound closure rates 48 h post-scratch, demonstrating a substantial enhancement of migratory capacity following overexpression of hsa-PHACTR4_0009 (n = 3, adj. p-value<0.0001 for each group, two-way ANOVA for multiple comparisons with Sidak's correction)**.**

We performed a wound healing assay in order to experimentally validate these transcriptomic findings. Over the course of the observation points at 0 h, 6 h, 12 h, 24 h, 48 h, monolayers of cardiomyocytes overexpressing circ-PHACTR4 showed significantly faster wound closure than vector control cells, suggesting enhanced ability to migrate (Fig. 5a, b). When compared to controls, the circ-PHACTR4 group's wound closure percentage at 48 h after scratch was significantly higher, according to quantitative analysis by Image J (Fig. 5c, d). All of these findings show that cardiomyocyte migration is enhanced by circ-PHACTR4 overexpression.

## 4 Discussion

d-TGA is the second most frequently occurring cyanotic CHD with an incidence rate of 0.2–0.3/1000 live births, and with a male-to-female ratio of 2:1 [18]. Despite advancements in genomics, transcriptomics, and proteomics, the molecular aetiology of d-TGA is still not understood. Interactions between multiple susceptibility genes [[19], [20], [21]] and environmental factors [22,23] are likely to cause complex conotruncal defects like Tetralogy of Fallot (TOF), d-TGA, and Double outlet Right ventricle (DORV), but the exact mechanism remains elusive. This suggests the role of epigenetics in the pathogenesis of such complex diseases, which may bridge the gap between multifactorial genetics and environmental influences. Various non-coding RNAs, such as microRNA (miRNA) and long non-coding RNA (lncRNA), have been explored as underlying principles behind the molecular pathogenesis of multiple diseases. Circular RNA, a recently discovered domain of non-coding RNA, plays critical roles in controlling the expression of various genes [24]. They are gaining attention in disease pathogenesis research due to the 5′-3′ covalently closed loop conferring unique structural stability and functional versatility, which makes them potentially better candidates for biomarker discovery, disease pathogenesis, and therapeutic intervention [25].

In our study, d-TGA neonates showed a significant decline in observed oxygen saturation, and a corresponding increase in the haemoglobin levels in the body of TGA patients was observed compared to healthy neonates. This may reflect the effect of compensatory mechanisms in response to cyanosis and lack of oxygen due to severe oxygen deficiency in TGA patients. Our results revealed that a circular RNA hsa-PHACTR4_0009 gets upregulated in the d-TGA condition, and it has a good prognostic potential, as evident from the ROC curve of hsa-PHACTR4_0009 with an AUC value of 0.9. and a likelihood ratio (LR) 4.250. An LR value >1 shows the association of the illness with the positive test results. LR compares the probability of a prognostic test result in a disease to the probability of the same result without the disease, directly indicating the diagnostic accuracy of a biomarker [26]. Moreover, the sensitivity and specificity of hsa-PHACTR4_0009 in detecting TGA cases were also good, indicating its utility as a prognostic tool for TGA in clinics. Furthermore, the expression levels of hsa-PHACTR4_0009 were associated with the degree of illness. In patients with normal heart anatomy, i.e., intact interventricular and/or interatrial septum and physiological closure of the ductus arteriosus following birth, the disease is severe because of poor mixing of blood in the left and right cardiac chambers. In such cases, the expression of hsa-PHACTR4_0009 was higher as compared to d-TGA complemented with ASD or VSD, suggesting the critical involvement of hsa-PHACTR4_0009 in the aetiology of d-TGA.

Next, to investigate the pathogenesis of d-TGA, we explored the predicted role of hsa-PHACTR4_0009 in any disease or molecular pathway. While the function of hsa-PHACTR4_0009 is not yet discovered, the parent gene of circular RNA hsa-PHACTR4_0009, i.e., PHACTR4 (Phosphatase and Actin regulator4), is reported to regulate cell migration of enteric neural crest cells (ENCCs) during embryonic development by remodelling actin cytoskeleton and activating protein phosphatase 1 [27]. This indicates the plausible role of its circular transcript hsa-PHACTR4_0009 in regulating the dynamics of the actin cytoskeleton during neural crest cell movements in embryonic development. Neural crest cells are crucial for the septation of the cardiac outflow tract (OFT) into the great arteries during cardiac development. Any disruption in NCC migration may lead to conotruncal defects such as d-TGA. Moreover, due to defective cell migration, dysregulated epithelial-to-mesenchymal transition (EMT) of endocardial cells may also lead to misaligned OFT, developing conotruncal anomalies such as d-TGA [27,28]. These findings led us to hypothesise that d-TGA arises due to defects in cell migration, which may be caused by circular RNA hsa-PHACTR4_0009.

The differentially expressed genes in our RNA-seq data between d-TGA cases and healthy controls were analysed to support our hypothesis. Significantly upregulated genes fell into focal adhesions and cadherin binding GO categories, the central signalling hub and the sites for cell connection to the extracellular matrix [29] and regulate the key processes of cardiac morphogenesis, i.e., cell migration, proliferation, and differentiation [30]. This highlights abnormal tissue integrity, impaired cell migration, and defective epithelial-mesenchymal transition (EMT) as the primary driving forces behind the developmental defects like d-TGA. Some genes with increased expression in d-TGA neonates included *VIM, PFN1, ACTG1, ARF-6,* and *EMD.*

*VIM* gene encodes an intermediate filament protein called Vimentin, which has a well-established role in cell migration and EMT by acting as a scaffold guiding focal adhesions and maintaining nuclear integrity to facilitate cell movements [[31], [32], [33], [34]]. Another protein, Profilin encoded by *PFN1,* is the G-actin binding protein which contributes to the leading edge of cell protrusions during cell motility and controls actin turnover during polymerisation [35,36]. Similarly, *ACTG1* encoding Gamma actin provides a structural framework to regulate cell migratory machinery through ROCK signalling and has been implicated in various diseases like cancer [37]. Studies have reported that overexpression of *ACTG1* enhances cell migration [38]. But its knockdown inhibits cell movements and proliferation [39]. *ARF-6* is another gene upregulated in d-TGA cases and is well recognised in maintaining cell shape and migration by regulating endocytic recycling and trafficking of cell adhesion molecules [40]. *Emerin* (*EMD*) maintains nuclear integrity and induces front-rear polarity of moving cells by asymmetric distribution of nuclear components during cell migration [17,[41], [42], [43]]. These evidences suggest that the defects in actin dynamics and neural crest cell migration may be the leading cause of reversal in the position of great vessels during embryonic development. In support of our hypothesis, we also observed enhanced cell migration with the overexpression of hsa-PHACTR4_0009, which possibly leads to abnormal migration of the cells, disturbing the architecture of OFT and great vessels during heart development. Additionally, the precise rotational and positional cues necessary for outflow tract remodelling are probably disrupted by excessive or uncoordinated migration, which leads to the aorta and pulmonary artery emerging from the wrong chambers. As a result, our study reveals abnormal cell migration as one of the main pathogenic mechanisms causing the formation of d-TGA.

## 5 Conclusion

Our study reveals the significant upregulation of a circular RNA hsa-PHACTR4_0009 in d-TGA compared to matched controls. Furthermore, hsa-PHACTR4_0009 may serve as a putative non-invasive prognostic biomarker for d-TGA because of its good sensitivity and specificity. As a regulator of actin dynamics and protein phosphatase 1, the Phactr4 gene encodes a circular RNA hsa-PHACTR4_0009, which might be involved in regulating cell movements during embryonic development. In support of this, we showed the key upregulation of genes like *Vimentin, Profilin, Gamma-actin, ADP ribosylation factor-6, and Emerin,* which are actively involved in cell migration, also getting upregulated in d-TGA cases. Perturbation in neural crest cell migration is reported to cause defects in the septation and the spiral rotation of OFT, which switches the morphological positions of aorta and pulmonary trunk. To confirm our findings, we also observed excessive cell migration with the overexpression of hsa-PHACTR4_0009, which may cause abnormal remodelling of great vessels. Therefore, our study highlights the enhanced cell migration as a direct cause underlying d-TGA development. More research is needed in the same direction to explore the molecular mechanism of d-TGA pathogenesis in detail.

## 6 Limitations of the study

Despite the promising insights into the molecular pathogenesis of d-TGA, our study has a few limitations. The sample size is the primary limitation, which may constrain the biomarker's statistical power and prognostic utility; more clinical studies with diverse patient cohorts are needed in this aspect. Secondly, our findings involving a significant upregulation of hsa-PHACTR4_0009 and the genes involved in cell migration are correlative and do not establish a direct causal association of hsa-PHACTR4_0009 with the development of d-TGA phenotype. Furthermore, functional validation through in vivo assays is needed to confirm the mechanistic role of hsa-PHACTR4_0009 in cell migration. Lastly, the expression of other regulatory RNA or proteins interacting with hsa-PHACTR4_0009, comprising a competing endogenous (ceRNA) network, has not been explored. This may furnish a more comprehensive understanding of the molecular mechanisms involved in d-TGA.

## CRediT authorship contribution statement

**Neha Rawal:** Writing – original draft, Validation, Investigation, Formal analysis, Data curation. **Anmol Gurgela:** Methodology, Data curation. **Snigdha Kumari:** Supervision, Funding acquisition, Data curation. **Manoj Kumar Rohit:** Funding acquisition, Data curation. **Ajay Bahl:** Supervision, Funding acquisition, Data curation. **Anupam Mittal:** Writing – review & editing, Supervision, Software, Project administration, Funding acquisition, Formal analysis, Data curation, Conceptualization.

## Declaration of competing interest

The authors declare the following financial interests/personal relationships which may be considered as potential competing interests: \ Anupam Mittal reports a relationship with Post Graduate Institute of Medical Education and Research, Chandigarh that includes: employment. I am not involved in the editorial process. If there are other authors, they declare that they have no known competing financial interests or personal relationships that could have appeared to influence the work reported in this paper.

## Appendix A Supplementary data

Supplementary material Image 1
