# Supplementary material for: Identification of circular RNA hsa-PHACTR4_0009 as a new class of biomarker for transposition of the great arteries
Source: J Mol Cell Cardiol Plus. 2025 Nov 23;14:100830. doi: 10.1016/j.jmccpl.2025.100830 (PMC12704311; doi:10.1016/j.jmccpl.2025.100830)
Supplement: Supplementary file 1 — Supplementary material [file mmc1.docx]

**Supplementary data**

**Identification of differentially expressed circular RNA in the d-TGA and control groups.**

Whole transcriptomics with circular RNA enrichment analysis revealed differentially expressed genes (DEGs). Using CIRCexplorer2v2.3.8, alignment and annotation of circular RNA were done, and the workflow pipeline to predict circular RNA (Figure S1). PCA plot revealed 25.82% of total variance in the first principal component (PC1) and 39.12% of total variance in the second principal component (PC2) (Figure S2). This indicates that control and d-TGA samples were clustered in PC1, showing similar impacts and indicating their intra-group resemblance. Moreover, PC2 revealed specific differences in the circular RNA expression profiles, indicating inter-group differences between the control and d-TGA samples.

**Figures**


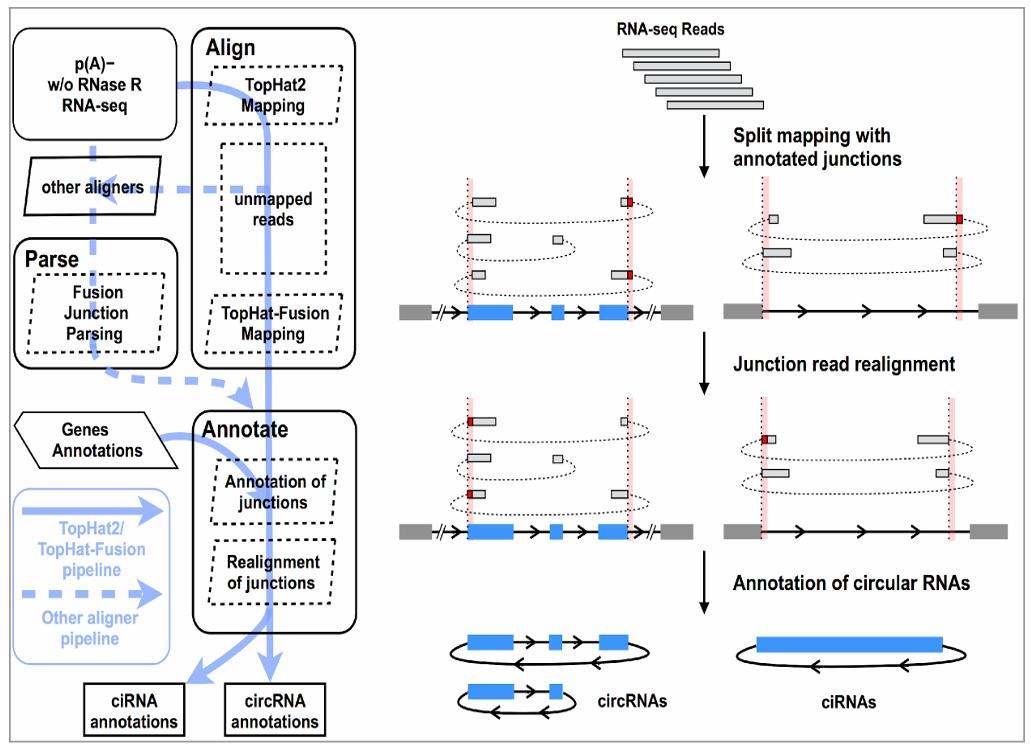


Figure S1. Pipeline for the prediction of circular RNA


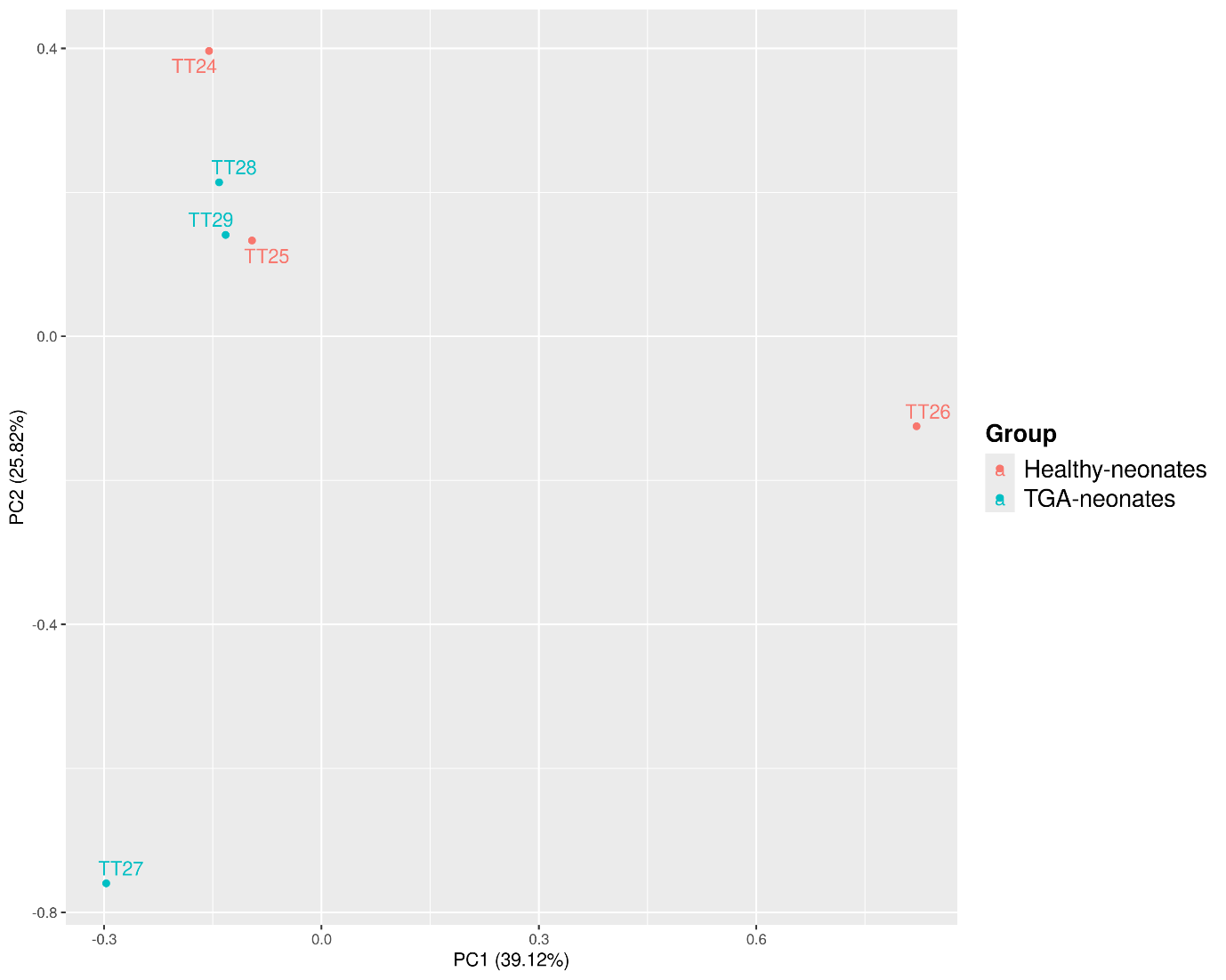


Figure S2: PCA plot representing intra-group samples clustered but distinct from the inter-group samples.

**Primer Sequences:**

| Name | Forward Primer | Reverse Primer |
| --- | --- | --- |
| hsa-circPHACTR4 | CACAGTGTCTACGGGAGGAA | GGGTGGGGACGGTTTTGATA |
| hsa-circMBOAT4 | TCATAGGAGTGGAGAACATGCA | AGTGCAAGATAAAGGCCCAA |
| hsa-GAPDH | GAGTCCACTGGCGTCTTCA | GGTCATGAGTCCTTCCACGA |
